# Diverse interactions of *Mycobacterium tuberculosis* infection and of BCG vaccination with SARS-CoV-2

**DOI:** 10.3389/ftubr.2024.1378068

**Published:** 2024-04-23

**Authors:** Padmini Salgame, Sri Ram Pentakota, John Carlo M. Malabad, Prakash Babu Narasimhan, Sheetal Verma, Senbagavalli Prakash Babu, Vartika Sharma, Sonali Sarkar, Marissa M. Alejandria, Jerrold J. Ellner

**Affiliations:** ^1^Department of Medicine, Center for Emerging Pathogens, Rutgers-New Jersey Medical School, Newark, NJ, United States; ^2^Department of Medicine, Rutgers New Jersey Medical School, Newark, NJ, United States; ^3^Institute of Clinical Epidemiology, National Institutes of Health, University of the Philippines Manila, Manila, Philippines; ^4^Department of Science and Technology, DOST Compound, Taguig, Philippines; ^5^Department of Clinical Immunology, Jawaharlal Institute of Postgraduate Medical Education and Research, Puducherry, India; ^6^Department of Preventive and Social Medicine, Jawaharlal Institute of Postgraduate Medical Education and Research, Puducherry, India

**Keywords:** SARS-CoV-2, tuberculosis, trained immunity, COVID-19, *Mycobacterium tuberculosis*

## Abstract

The COVID pandemic and tuberculosis (TB) endemicity is double trouble to much of the world. SARS-CoV-2 and *Mycobacterium tuberculosis* (Mtb), causative agents of COVID and TB, respectively, are both infectious respiratory pathogens involving close communities and individuals. Both pathogens can cause lung disease, involving unbalanced inflammatory cell immune responses that can lead to a syndemic impact. Moreover, dual infection is common in certain settings. In low- and middle- income countries, most individuals with SARS-CoV-2 infection or COVID-19, in fact, will have been exposed to or infected with Mtb and some will develop active TB. Here we review the literature examining the diverse interactions of *M. tuberculosis* infection and of BCG vaccination with SARS-CoV-2. We discuss areas in which contradictory results have been published and conclude that there are still several unresolved issues that warrant further study on the co-pathogenesis of SARS-CoV-2 and Mtb and BCG- mediated heterologous protection against COVID-19.

## Introduction

The clinical, epidemiologic, and biologic interactions of *Mycobacterium tuberculosis* (Mtb) infection (MTBI), and of BCG vaccination with SARS-CoV-2 infection and may be substantial, diverse and bidirectional. SARS-CoV-2 and *Mycobacterium tuberculosis* (Mtb), causative agents of COVID and TB, respectively, are both infectious respiratory pathogens involving close communities and individuals. Both pathogens can cause lung disease, involving unbalanced inflammatory cell immune responses that can lead to a syndemic impact. (1). Moreover, dual infection is common in certain settings. In low- and middle- income countries, most individuals with SARS-CoV-2 infection or COVID-19, in fact, will have been infected with Mtb by adolescence (2, 3) and some will develop active tuberculosis (TB). COVID-19 also may occur before TB particularly with the nearly ubiquitous frequency of SARS-CoV-2 in the population (3, 4). Therefore, it is important to consider interactions that may be impacted by the order of acquisition of these pathogens and the stage and severity of the resultant infection and disease.

There may be relevant lessons from the interactions of Mtb and HIV-1 (5). The pandemic of HIV-1 infection had profound impact on TB (6). A surge in new cases of TB was the harbinger of a new, at the time, unknown infectious agent spreading in Africa. HIV-1 affected the natural history, manifestations, and course of TB. It soon became apparent that TB also affected the course of HIV-1 infection, promoting viral replication and accelerating progression to immunodeficiency (7, 8). The impact on TB on other co-infections such as malaria, Middle East respiratory syndrome (MERS), and severe acute respiratory syndrome (SARS) has been addressed (5, 9–11).

There are similarities and overlap in the pathogenic mechanisms of Mtb and of SARS-CoV-2. Each is spread by the aerosol route and engages receptors prominent in type II pneumocytes, making the lungs the primary initial target of infections. SARS-CoV-2 binds to the receptor-binding domain (RBD) of angiotensin-converting enzyme 2 (ACE2) (12); and although alveolar macrophages are recognized as the primary host entry point for Mtb (13, 14), the pathogen also targets type II pneumocytes (15). Another similarity is the activation of Type I interferons (IFNs). The role of IFNs in driving TB pathogenesis is well-described (16–18). Likewise, IFNs stimulate the expression of SARS-CoV-2 entry receptors and mediate endocytosis of the virus (19). Given the strong evidence of the involvement of IFNs in the pathogenesis of both TB and COVID-19 (20, 21), the impact of heightened IFNs may be detrimental to the host. Leukopenia and lymphocytopenia are hallmarks of both TB and COVID-19 with concomitant perturbation in the numbers and function of T cells, B cells, NK cells and neutrophils (22–24). These similarities in pathogenesis suggest that there may be synergistic impact of co-infection on immunopathogenesis. As of December 2023, PubMed lists 1,830 publications on TB and COVID-19. We are aware of only two general reviews of the interaction, one published early in 2021 and the other focused on immune mechanisms (25, 26). We will discuss various facets of SARS-CoV-2 and Mtb interactions subsequent outcomes in the co-infected host based on the currently available data.

## Impact of SARS-CoV-2 on TB disease

### Public health/epidemiology

The pandemic of SARS-CoV-2 destabilized TB control programs because personnel and laboratory resources were shifted to address the COVID-19 pandemic (27). As a result, the diagnosis of TB was delayed. TB treatment completion was affected, as well, by reduced mobility of both patients and health care professionals. This was superimposed on a general decline in health care access and an increase in poverty (28). The WHO Global TB Report released in October 2022 (29), showed a decrease in the number of persons reported with TB from 7.1 million in 2019 to 5.8 million in 2020 (18%) and 6.4 million in 2021; there was a major global recovery in 2022 with 7.5 million reflecting a “back-log” (30). In fact, deaths attributed to TB were 1.4 million in 2019, 1.5 million in 2020; 1.6 million in 2021 and 1.4 million in 2022. The COVID-19 pandemic was estimated to cause 500,000 additional deaths from TB. Other consequences of the pandemic were a 17% decline in people provided treatment for drug resistant TB, a 21% decline in provision of preventive therapy. and a decrease in global spending for essential TB services. COVID-19 also was associated with increased food insecurity worldwide; the attendant malnutrition no doubt will further increase TB prevalence in countries with a high TB burden (31). A recent thematic scoping review addressed the global public health impact of COVID-19 on TB including discussions of mental health and stigma (32).

### Clinical manifestations

Co-infection with TB and COVID complicates the diagnosis and management of each because of similar transmission routes, clinical symptoms and involvement of the lungs (33, 34). Pulmonary disease and pathology are a hallmark of acute COVID-19 and usually takes the form of viral pneumonia and adult respiratory distress syndrome (ARDS). Patients with COVID-19 are at risk for additional lung damage due to subsequent infections with other respiratory pathogens including TB. SARS-CoV-2 infections also are associated with hyperinflammation and multi-system inflammatory syndrome. Theoretically, there could be a failure to modulate the inflammatory response in TB co-infection with more extensive lung involvement and damage. COVID-19 patients with hyperinflammation may be treated with immunosuppressive treatment such as dexamethasone and Tocilizumab (monoclonal antibody against IL-6 receptor) which may in turn promote progression from Mtb infection to disease. In addition to TB prevalence these immunosuppressive therapies may well-impact TB presentation (late-stage presentation/more severe disease), treatment outcomes: i.e., increased mortality and treatment failure, and TB sequelae: i.e., more post TB pulmonary dysfunction. In the first published report from the Global Tuberculosis Network of 49 patients with TB and COVID-19 (35), 43 (88%) were symptomatic and hospitalized, 20 needed oxygen supplementation and six died. In this cohort (35), 26 (53%) had TB diagnosed before COVID-19, seven of whom had post-TB pulmonary sequelae. Fourteen cases (28.5%) had COVID-19 first and nine (18.3%) had both diseases diagnosed within the same week (35). Pulmonary, compared to extra pulmonary TB was predominant in co-infected cases as in TB alone; both drug-susceptible and drug-resistant mycobacterial strains were reported equally (35). Two cohort studies indicated that co-infection was more common in migrants and in males (35, 36). Among patients with TB and COVID-19 both unilateral pulmonary and bilateral infiltrates have been reported (35, 36). A meta-analysis of 20 studies, with a total of 205,702 patients found that patients with TB had an increased risk of mortality during a co-infection with SARS-CoV-2 (37). This may be explained, in part, by the reduced frequency of Mtb-specific CD4 T cells in COVID-19 patients (38).

### Treatment outcomes

COVID-19 had a moderate effect on the prognosis and clinical response to antituberculosis treatment (ATT) in the short term (39). In a systematic review that examined the impact of COVID-19 co-infection with TB there was, however, no significant association between SARS-CoV-2 coinfection and un-favorable TB treatment outcomes (40). Completion of the long-term outcome evaluations of the global TB and COVID-19 co-infection cohort of 767 patients found that death was the outcome in >10% of the coinfected patients (41). Furthermore, among survivors, success of TB-treatment was reduced (41). A retrospective cohort study conducted in New York City reported significantly higher mortality risk over a 2-year period among those diagnosed with TB/COVID-19 within 120 days of each other compared to those diagnosed with TB alone (adj. HR 2.69, 95% CI: 1.66–4.13) (42). Age-adjusted mortality rates were higher among California residents diagnosed with TB/COVID-19 (74.2 per 1,000 persons) compared to those diagnosed with TB during the pre-pandemic period (56.3 per 1,000 persons) (43). An important finding in the global co-infection cohort was that greater numbers of COVID-19 cases did not recover if SARS-CoV-2 was acquired after the end of TB treatment (26.8%) than in those with COVID-19 diagnosis before or during TB treatment (10.3%) (41, 44). This suggests that post-TB lung disease negatively impacts COVID-19 disease outcome.

## Impact of SARS-CoV-2 on MTBI

COVID-19 may promote reactivation of TB by triggering type 1 IFN signaling which is permissive for mycobacterial growth (45) and through local effects such as lung inflammation and fibrosis (46, 47). Immunosuppression caused by SARS-CoV-2 infection could lead to activation and progression of existing TB foci (33) and progression from MTBI to TB. There is a decrease in the numbers as well as functional exhaustion of T cells in response to COVID-19 infection (48) and COVID-19-induced cytokine storm (49). Rajamanickam et al. (50), in fact, found increased baseline and Mtb antigen induced cytokine responses in persons with MTBI in the presence of SARS-CoV-2 seropositivity. Despite biologic plausibility there are no direct data concerning how often SARS-CoV-2 leads to TB. In addition to direct effects of SARS-CoV-2 infection, corticosteroids administered to treat COVID-19 patients creates an immunosuppressive state and an opportunity for reactivation of latent TB (51, 52). Treatment of COVID-19 may attenuate inflammatory processes required for host containment of MTBI (1, 53). Case reports have described activation of latent TB to active TB and development of TB in patients with no history of exposure to TB during their post-COVID period (54, 55), as seen in past viral pandemics (56). In one instance, a 40 year old female with possible latent TB developed active tuberculosis 7 weeks after her initial infection with COVID-19 (57). In another report, a 29-year-old healthy male with no prior exposure or history to Mtb, was diagnosed with miliary pulmonary TB after COVID-19 infection. The prolonged usage of corticosteroids for the treatment of COVID-19 can lead to reactivation of TB and also reduces the permeability of anti-TB drugs into lung epithelium impacting their effectiveness (58).

## Impact of TB on SARS-CoV-2

In the global cohort described above of 767 TB/COVID-19 co-infected patients from 34 countries covering the period from March 2020 to June 2021, the mortality rate was 11.1% and hospitalization due to COVID-19 was 61.7% (59). The authors suggest that TB, either active or inactive, is an important risk factor for the development of more severe forms of COVID-19 disease. In an updated meta-analysis that included 36 studies and 60,103 COVID-19 patients from Asia, Africa, Brazil and USA, covering the period of January 2020 to May 2021, the authors found an increased risk for severe COVID-19 infection (OR = 1.56, 95% CI: 1.13–2.16) and death (OR = 1.94, 95% CI: 1.28–2.93) among patients with TB compared to those without TB (60). Therefore, TB is a significant risk factor that increases morbidity and mortality among COVID-19 patients.

Although experimental data on the immunopathology of TB/COVID-19 co-infection are limited, TB (past or current) induces lung damage by the proinflammatory response in the lung parenchyma that, in turn, may increase susceptibility to other airborne pathogens, such as SARS-CoV-2. The immune responses implicated in TB and COVID-19 pathogenesis are similar, involving local expression of TNF and IFNγ, cytokines which contribute to inflammation and accumulation of active cells in the lung. During TB and COVID-19 co-infection, inflammatory stimuli may add up, leading to further lung tissue damage (1, 61). A systemic manifestation of TB/COVID-19 co-infection is T-cell exhaustion. The Th-1 immune response intensified by SARS-CoV-2 infection in patients with pre-existing TB may cause depletion and dysfunction of T-cells (CD4+ and CD8+), and immune dysregulation increased expression of pro-inflammatory cytokines and cytokine storm (62) potentially acute disease and long-term sequelae. Another mechanism by which TB may increase the susceptibility to and severity of COVID-19 is through activation of myeloid-derived suppressor cells (MDS), the known suppressors of T cells in viral infections (25). Further, in cases of TB with cavitary lesions, the distorted pulmonary architecture could result in increased susceptibility to SARS-CoV-2-induced pneumonia and respiratory failure. As a consequence, patients with co-infection have severe disease and poorer prognosis (1).

## Impact of MTBI on SARS-CoV-2

MTBI may itself be associated with immune activation (63–65) providing a potential mechanism for co-pathogenesis, and, further, inflammation may be prolonged following an episode of TB despite apparent clinical “cure” (66). Regarding the relationship between latent TB infection (LTBI) and COVID-19, a study which used observational case-control design involving 36 TB/COVID-19 patients from multiple primary care hospitals in China reported that COVID-1 was twice as likely to occur in those with latent TB infection (LTBI)+, determined through interferon-gamma release assay (IGRA), compared to the general population (61). This finding suggests that LTBI was a risk factor for susceptibility to COVID-19. There also was a significantly higher proportion of LTBI among severe and critical COVID-19 cases compared to those with mild and moderate disease (78 vs. 22%; *p* = 0.0049). Studies from the Philippines and the Global Tuberculosis Network also suggested a potential increase in susceptibility to SARS CoV-2 and increase in COVID-19 severity among patients with active or past TB or LTBI (35, 67). These cohort studies however lacked adjustment for potential confounders such as comorbidities.

There is a theoretic possibility, in fact, that LTBI may protect against or ameliorate SARS-CoV-2 infection through trained immunity. Trained immunity is defined as epigenetic and metabolic reprogramming of innate immune cells leading to enhanced non-specific antimicrobial response to a secondary infection (68, 69). This phenomenon as manifest in monocytes of individuals recently exposed to Mtb (70) conceivably would lead to heterologous protection and protection against or a milder course of SARS-CoV-2 infection. In a study conducted in India, severely ill patients with COVID-19 were less like to be IGRA+ although it was not possible to discern whether IGRA+ status was protective or severely ill patients lost their IGRA response (71). In a study of 76 patients with COVID-19 in Turkey a positive tuberculin skin test (TST) was associated with milder disease (72); again it is not clear whether COVID-19 suppressed the TST response. These results are contradictory to those reported from China and the Philippines as discussed above and raise the possibility that LTBI under certain circumstances may compromise the induction of trained immunity. In this regard, a study performed in the mouse model showed that Mtb indeed impaired the development of protective trained immunity by uniquely reprogramming haematopoietic stem cells (HSCs) via type I interferon (73). Despite its ability to repress trained immunity, Mtb infection induced resistance to secondary infection with SARS-CoV-2 in two different mouse models, (K18-hACE2 (ACE2) mice infected with SARS-CoV-2 and C57BL/6 mice infected with a mouse-adapted SARS-CoV-2) (74). Clearly, additional studies are required to fully comprehend the impact of LTBI induced host immune response on SARS-CoV-2 infection.

## Bacillus Calmette-Guérin and heterologous protection against COVID-19

There is evidence, as well, that BCG vaccination of infants protects them from pathogens other than Mtb resulting in heterologous protection (75). Subsequent studies showed that vaccine-induced heterologous immunity was mediated by durable epigenetic modifications to the innate immune response, ergo, trained immunity (76, 77). In BCG-vaccinated adults, circulating monocytes and NK cells acquired a trained phenotype, characterized by increased production of proinflammatory cytokines (68, 69). Members of the IL-1 family play an important role in trained immunity (78). Importantly, a randomized placebo-controlled human challenge study showed that BCG-induced trained immunity provided protection *in vivo* in a pathogen agnostic manner (79). Subjects were challenged with attenuated yellow fever virus vaccine strain and the BCG-vaccinated showed lower viremia compared to controls. The decreased viremia strongly correlated with genome-wide epigenetic reprogramming of human monocytes and associated increased IL-1β production (79). Mechanistically, BCG induces trained immunity by reprogramming haematopoietic stem cells (HSCs) in the bone marrow via a type II interferon pathway (80).

These findings provided the rationale for studies of whether BCG-induced trained immunity could serve as a tool against COVID-19 (81, 82). Early in the COVID-19 pandemic, several observational studies and small clinical trials suggested that trained immunity induced by BCG vaccination might have a protective effect against SARS-CoV-2 infection. For example, in a study from Turkey the decreased mortality in health care workers with COVID-19 might have been due to increased Mtb exposure history and BCG vaccination (83). In a unicentric, randomized-controlled clinical trial, revaccination of health care workers with BCG Moscow was associated with a lower incidence of COVID-19 positivity, though the results did not reach statistical significance (84). Prior BCG vaccination of health-care workers was associated with decreased SARS-CoV-2 IgG seroconversion (85).

Sixty-five randomized controlled trials of BCG to prevent or ameliorate COVID-19 have been registered to date. In a phase 3, randomized, double-blind, multicenter clinical trial in healthy elderly volunteers, vaccination with VPM1002, a genetically modified BCG, prevented severe respiratory disease including that due to COVID-19 (86).

However, data from recent clinical trials fail to show a protective role for BCG against SARS-CoV-2 infection. Meta-analyses of 7,963 participants from nine randomized controlled trials as of November 2022, showed no efficacy of BCG against COVID-19 infection (OR, 0.96; 95% CI: 0.82–1.13); COVID-19 related-hospitalization (OR, 0.66; 95% CI: 0.37–1.18); ICU-admission (OR, 0.25; 95% CI: 0.05–1.18) or mortality (OR, 0.64; 95% CI: 0.17–2.44) (87). Two, more recent, randomized, placebo-controlled trials in healthcare workers (BCG-Corona Study Group and BRACE Trial Consortium Group) showed that BCG vaccination did not reduce SARS-CoV-2 infections, symptomatic COVID-19 or severe COVID-19 in individuals with sero-confirmed infections as well as self-reported positive SARS-CoV-2 tests (88, 89). Further, BCG vaccination, despite enhancing cytokine and antibody responses to SARS-CoV-2, had no effect on the incidence of SARS-CoV-2 infection in older adult volunteers (90). Nor did BCG protect health-care workers in South Africa from SARS-CoV-2 infection or related severe COVID-19 disease and hospitalization (91). During the COVID-19 pandemic, BCG-vaccination of HCW exposed to COVID-19 patients did not reduce unplanned absenteeism nor documented COVID-19 (92). In a multi-center, randomized, double-blind, placebo-controlled study on a group of 695 health care workers aged 25 years and over in Poland, statistical analysis did not reveal any significant correlation between the frequency of incidents suspected of COVID-19 and BCG-10 vaccination, the result of the tuberculin test or the number of BCG scars (93).

There seems to be a disconnect between the early observational studies and experimental models and the results of randomized clinical trials. Possible explanations are: (i) inclusion of trial participants with active TB and LTBI; (ii) variance in protective efficacy and immunogenicity across BCG strains; (iii) age and gender driven differential non-specific effects of BCG; and (iv) impact of recent vaccination history (94, 95). An important factor to be considered is whether BCG was administered at birth so that the trial dose represented a vaccine boost; and also, that efficacy may vary against individual SARS-CoV-2 strains. BCG vaccination could, in fact, be effective in certain populations. In a high-risk population, a multi-center quadruple-blind study showed that BCG had protective effect and reduced the incidence of acute respiratory illness in symptomatic COVID-19 infection and the severity of the disease (96). In a randomized, double-blinded, placebo-controlled phase 2/3 trial conduced in the USA, the safety and efficacy of a multi-dose (BCG) vaccine for the prevention of COVID-19 was evaluated in a COVID-19-unvaccinated population of adults with type 1 diabetes. The study found that BCG vaccinated group had fewer cases of confirmed COVID-19 in comparison to the placebo group, with vaccine efficacy at 92% (97). The severity of COVID-19 was also significantly lower in the vaccinated group compared to the placebo. It is of interest that blood immunoglobulin G (IgG) levels specific to SARS-CoV-2 were higher in the BCG group, albeit without statistical significance (98). BCG also could have an immunomodulatory effect potentially increasing the efficacy of specific vaccines or prior SARS-CoV-2 infection.

In experimental models the efficacy of BCG against MTB is greater when administered intravenously (80, 99, 100). In mice, intravenous BCG immunization provided significant cross-protection against subsequent influenza A virus infection through the induction of trained immunity mediated by IFNγ secreted from CX3CR1^hi^ effector memory T cells (101). Intravenous BCG vaccination also elicited strong protection against SARS-CoV-2. For example, in the golden Syrian hamster model, intravenous BCG vaccination reduced viral load and SARS-CoV-2 severity (102). Intravenous administration of BCG also protected mice against lethal SARS-CoV-2 challenge (103, 104) via BCG-specific Th1 cell mediated prolonged innate antiviral resistance (104). Although there are unresolved issues, the impetus to use BCG vaccine for non-specific protective effects should be pursued, especially given that the iv approach can be used with the development of “suicide” BCG strains that are cleared in immunocompetent and immunocompromised hosts (105).

## Conclusions

**Figure 1:**
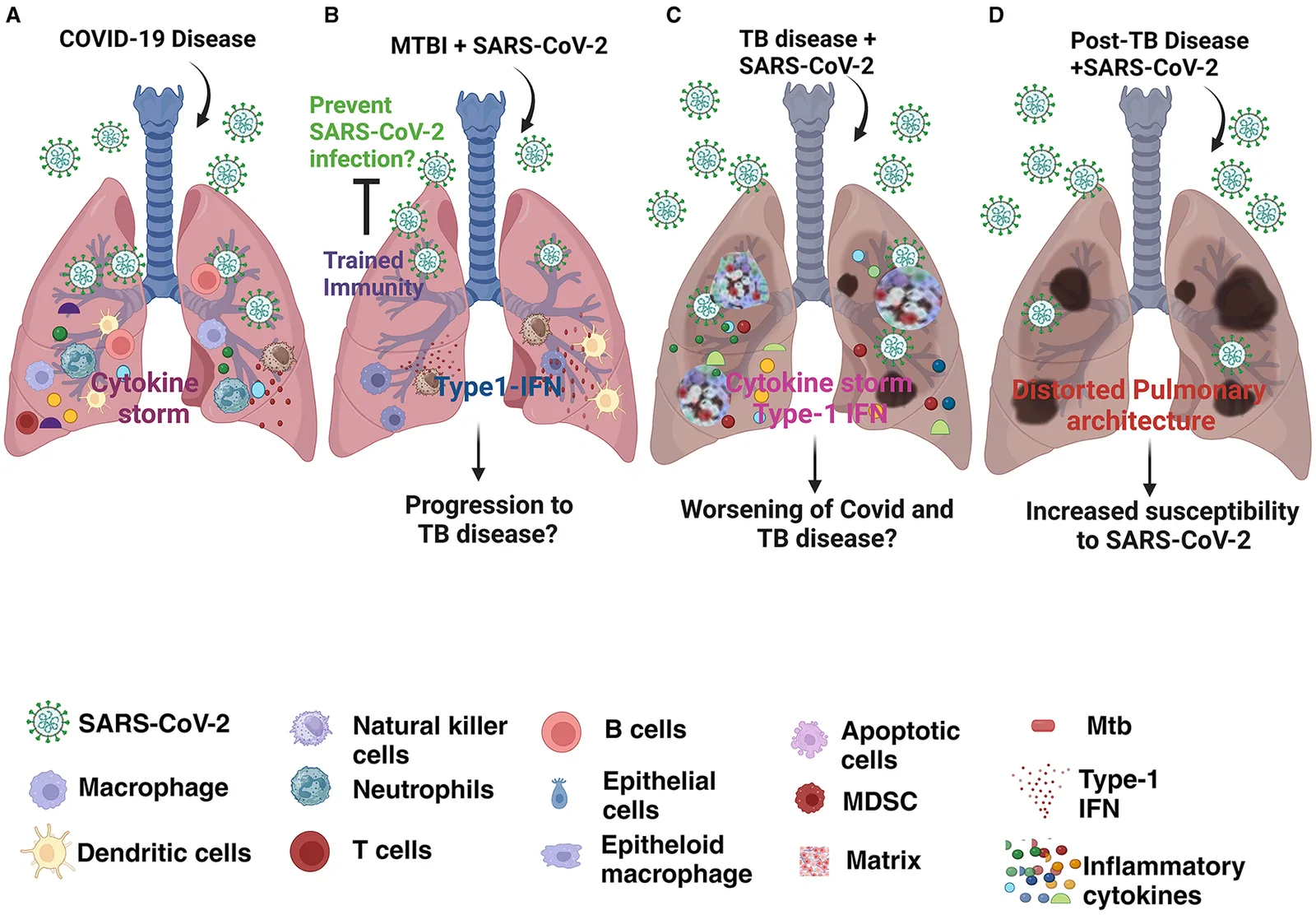
Schematic representation of lung pathology in **(A)** COVID-19 disease; **(B)** Mtb infected individual (MTBI) coinfected with SARS-CoV-2; **(C)** Individual with TB disease and SARS-CoV-2 coinfection; **(D)** SARS-CoV-2 infection in individual post-TB disease.

The available data as reviewed support synergism in the co-pathogenesis of TB and COVID-19 in dually infected persons (Figure 1). There are, however, several areas in which contradictory results have been published. The complexity of obtaining clearcut results is explained by considering that the natural history of infection with MTB and of SARS-CoV-2 each represents a continuum so that there is marked heterogeneity in the stage of co-infection at the individual level. There too are compounding effects of host demographics and co-morbidities, the infecting strain of SARS-CoV-2 and of Mtb and the level of protection conferred by prior COVID-19, vaccination and both. This leaves the possibility that several areas of uncertainty may never be resolved.

The impact of the COVID-19 pandemic on TB control measures and consequently on epidemiology and lethality of TB is clear-cut and well-documented. Also, the lung damage of both diseases is at least additive and may impact on short- and long-term outcomes including survival. The mechanism of co-pathogenesis appears to involve modulation of ACE on pneumocytes, Type 1 interferons, immune activation, inflammation and the cytokine storm. Although there is no evidence to date that SARS-CoV-2 vaccinations affects the incidence or prevalence of TB, it may modify co-pathogenesis.

**Figure 2:**
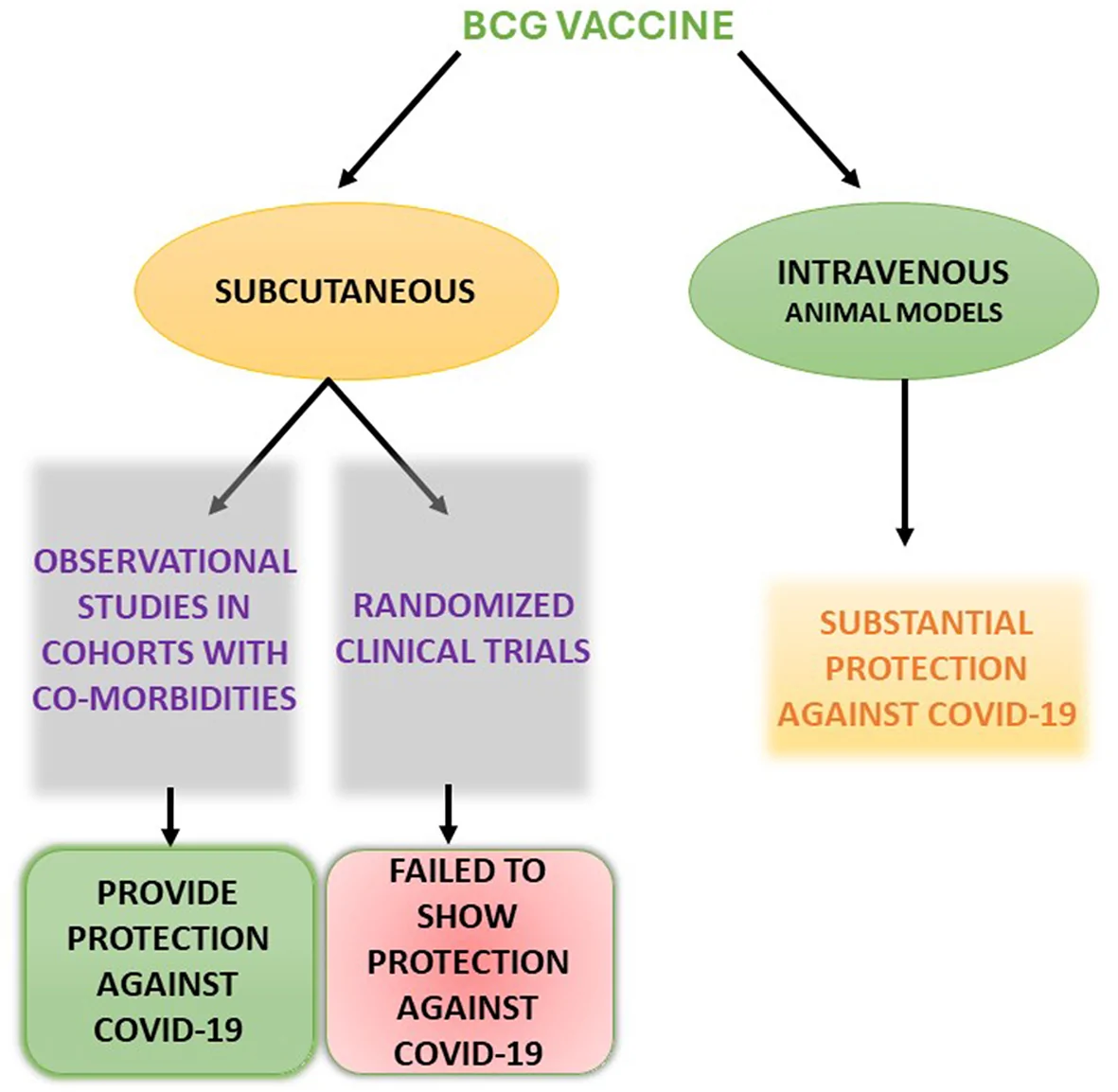
Schematic representation of the varied outcome of SARS-CoV-2 following BCG vaccination.

Perhaps the area of greatest uncertainty is whether MTBI or revaccination with BCG confers heterologous protection against SARS-CoV-2 (Figure 2). The evidence from *ex vivo* and experimental studies indicates that BCG vaccination confers training of the immune response and/or protection from heterologous challenge. The results of randomized controlled trials, however, have not shown that BCG revaccination impacts on the incidence or severity of COVID-19. The myriad of potential confounders is difficult to address. There may, however, be greater efficacy of BCG vaccination in subpopulations (high-risk groups, pre-existing Type 1 diabetes mellitus. It will be of interest to determine whether BCG modifies the immune response to other vaccines including SAR-CoV-2 vaccines. These unresolved issues assume additional importance as revaccination with BCG is under investigation for prevention of TB. The possibility that this approach will provide some level of non-specific protection against current and future pathogens is intriguing. Hopefully data from the trials that are completed or in progress will shed light on this issue.

## Author contributions

PS: Conceptualization, Funding acquisition, Writing – review & editing. SRP: Writing – original draft. JM: Writing – original draft. PN: Writing – original draft. SV: Writing – original draft. SP: Writing – original draft. VS: Writing – original draft. SS: Funding acquisition, Writing – original draft. MA: Funding acquisition, Writing – original draft. JE: Conceptualization, Funding acquisition, Writing – review & editing.
